# Promoting Open Discussions of Scientific Failure within the Annual Society for Neuroscience Conference

**DOI:** 10.1523/ENEURO.0524-24.2024

**Published:** 2025-03-04

**Authors:** Megan H. Hagenauer, A. David Redish, Daniela Schiller, Kristin L. Bigos, Shelly Flagel, Amilcar Rodriguez, Zac Parker, Angela O’Connor, Xilma Ortiz-Gonzalez, Deirdre Murphy, Rachel Leeson, Community for Rigor

**Affiliations:** ^1^Michigan Neuroscience Institute, University of Michigan, Ann Arbor, Michigan 48109; ^2^Department of Neuroscience, University of Minnesota Medical School, Minneapolis, Minnesota 55455; ^3^Departments of Neuroscience and Psychiatry and Friedman Brain Institute, Icahn School of Medicine at Mount Sinai, New York, New York 10029; ^4^Department of Medicine, Division of Clinical Pharmacology; Department of Pharmacology and Molecular Sciences; Department of Psychiatry and Behavioral Sciences, Johns Hopkins School of Medicine, Baltimore, Maryland 21287; ^5^Comparative Biomedical Sciences Program, North Carolina State University, Raleigh, North Carolina 27695; ^6^Community for Rigor, Philadelphia, Pennsylvania 19104; ^7^Division of Neurology, Children’s Hospital of Philadelphia, Philadelphia, Pennsylvania 19104; ^8^Cognitive Neuroscience, Florida International University, Miami, Florida 33199; ^9^AddGene, Watertown, Massachusetts 02472

**Keywords:** mentorship, perseverance, reproducibility, storytelling, transparency, troubleshooting

## Abstract

The annual Society for Neuroscience (SfN) meeting is a bonanza of scientific achievement: famous keynote speakers, beautiful scientific results, and award ceremonies. This focus is exciting and invigorating but glosses over the many failures, mistakes, and rejections that typically lead to scientific success. Our goal has been to create a space within the annual SfN meeting for open conversation about scientific failure and, by doing so, increase transparency, resilience, and mental well-being within our community. In this article, we share the materials that we have used at SfN during the past 4 years (2021–2024) to promote discussions of scientific failure, including formal storytelling, individual and interactive games, and confessionals. For each activity, we provide the rationale and practical guidance regarding logistics and usage. We hope this will aid scientists interested in adapting the activities for their own communities or local events. We end with a call for scientific institutions to commit to providing space for open discussions of failure within their educational programs and conferences.

## Significance Statement

Open discussions of failure can increase transparency, resilience, and mental well-being within the scientific community. In this article, we share the materials that we have used at the annual Society for Neuroscience (SfN) meeting (2021–2024) to promote open discussions of scientific failure, along with their rationale, and practical guidance regarding their logistics and usage. These materials can serve as a guide for scientists and scientific organizations interested in providing space for open discussions of failure within their educational programs and conferences.

## Introduction

The daily experience of science is often one of failure. Most experiments fail. Most papers are triaged. Most grants are rejected. And most job candidates never get an interview. Despite this, failure is rarely discussed in public. Instead, the long stumbling chain of failures that lead to scientific findings are hidden between the lines of publications and omitted from presentations. The annual Society for Neuroscience (SfN) conference epitomizes this paradox: the content of the conference is overwhelmingly focused on personal and experimental success told through the medium of carefully constructed posters, slick slide presentations, and polished stories. Troubleshooting and commiseration are relegated to the afterhours, in restaurants and pubs.

However, experimental failure is indispensable to the fabric of discovery and development—it is through troubleshooting experiments and the failure of beloved theories that science moves forward. As Stuart Firestein put it, “*Infallibility just doesn’t belong to science*” (Great Scientists, Great Failures, 2021a)*.* In neuroscience in particular, we regularly bear witness to all the ways that science doesn't go as planned during our audacious attempts to understand something so complex as the human brain. Even for the most skilled of scientists, it is virtually impossible to predict and avoid all confounding sources of noise, or to execute complex, multistep experiments without making at least one mistake. As a matter of course, we draw incorrect conclusions and build theories from incomplete information.

With full transparency and data release, alongside room for open discussion and revision, most misunderstandings eventually resolve as more data are collected, and results from diverse experiments are compared. Indeed, the ultimate goal of science is to be “intelligently wrong”—to constantly build upon and revise our theories with new insight and information. However, in a highly competitive environment, many scientists are afraid that fully disclosing inconsistencies, mistakes, and weaknesses will draw criticism from peers or funders. Placing a polished, digestible story at the center of our scientific discourse is important for science communication, and for the progression of science. But doing so also encourages the imperfection and complexity inherent to science to be buried within carefully worded results and supplemental material, inaccurately representing the scientific process and precluding self-correction. If our field is to move beyond the reproducibility crisis, we need greater transparency, and, with that, we need to normalize the admission of failure.

The experience of pursuing a scientific career is also fraught with many failures and few successes, especially within the current system for scientific funding, publication, and career advancement. Appreciating the complex interplay between luck and skill that underlies success in scientific practice today is difficult. Finding support to deal with rejected grants and papers, lost awards, and never-gotten interviews is critical to mental well-being, perseverance, and success in the modern scientific enterprise. Seeing that others have dealt with these negative experiences and persevered (and how they persevered!) can help alleviate the accompanying distress.

Within this context, public discussions of scientific failure are an important way to promote the mental health and retention of individual scientists. For trainees, lack of discourse about failure can produce an illusion that they, personally, are struggling more than those around them. Failure may feel like a weakness that needs to be hidden, causing isolation and depression, and reinforcing imposter syndrome. Worse, failure can feel like a final verdict on their potential as a scientist. The need for open discussion of failure and struggle can be particularly great for scientists from underrepresented groups, where perseverance in the face of adversity is essential and frequently faced without role models and peer support.

For the general public, our emphasis on a beautiful, polished story is equally dangerous, as it feeds into the misperception that science is the infallible production of facts instead of a method that progressively uses data to develop and support theories. Within this space, any ounce of controversy or revision creates anxiety that the process isn't working, or—worse—generates suspicions of a conspiratorial elite.

Most importantly, failure is a powerful teacher, and by keeping it in the shadow of scientific meetings, we are missing opportunities to learn from others' struggles. At the heart of every story about failure is an important lesson, painfully gained and strikingly memorable. Sharing these stories is training at its best.

Thus, starting in late 2018, we set out to generate activities and events within the SfN conference that promote open discussion of scientific failure with three **objectives:**

1. To celebrate the universality and importance of failure within scientific pursuits, promoting realistic expectations and the risk-taking necessary for innovation.
2. To encourage scientists to openly discuss failure, promoting transparency and reproducible science.
3. To provide a forum for learning from each other's mistakes, promoting personal resilience and faster evolution of the field.

These efforts manifested in five events hosted at the large annual SfN conference during 2021–2024, including a high-profile storytelling session (2021: “Oh Sh*t: Great Scientists Tell Stories About Their Greatest Failures”) and four interactive socials (2021: “Oh Sh*t” Break-out Session, 2022–2024: “*The Confound Hour: Let's Make Some Noise!”*). We share the materials below for facilitating core aspects of these events, along with their rationale, reflections on their utility, and practical guidance regarding the logistics and usage for scientists interested in adapting them for their own communities.

## Formal Storytelling Session: “Oh Sh*t: Great Scientists Tell Stories About Their Greatest Failures”

### Context

SfN has featured a formal storytelling session as part of the annual conference for almost a decade. These storytelling sessions are intended to humanize science and convey its importance in a compelling way. Our storytelling session “Oh Sh*t: Great Scientists Tell Stories About Their Greatest Failures” was featured as part of the 2021 SfN conference.

### Inspiration

We were inspired to run a storytelling session about scientific failure at SfN by a global movement within the entrepreneurial community called the “F*ckUp Night” (https://fuckupnights.com). At these events, powerful people share their personal stories of failure to pass on wisdom to younger members and encourage them not to give up in difficult careers.

### Vision

We hoped that listening to well-established scientists open up about their struggles and mistakes would reduce stigma and, ideally, initiate a ripple effect, inspiring conversations about failure throughout the rest of the conference, in socials and luncheons. We designed the event to follow a format akin to the live storytelling events hosted by The Moth, (n.d.), with each storyteller taking the stage, stepping into the spotlight, and—with just a microphone—diving into their personal story. No slide presentation, just raw honesty about their lived experience of doing science.

### Logistics

The storytellers were recruited via e-mail, social media, and word-of-mouth. We targeted scientists who were either at the top of their field or rising stars, with the goal of sending a clear message to the audience that even highly successful scientists make big mistakes. Due to the nontraditional format, we also looked for individuals who were successful as scientists in part due to their storytelling ability, or who were known to be eloquent, insightful, genuine, wry, or hilarious—individuals who were secure in their own skin or known for their introspection and frank honesty. We also sought individuals who had been intensively involved in the mentorship of younger scientists, winning teaching awards or serving as directors for local undergraduate or graduate programs.

Recruitment turned out to be remarkably challenging—even the most famous and confident scientists were (understandably) uncomfortable at the prospect of sharing their most vulnerable moments in a professional setting in front of a large audience of their peers, within a nontraditional, unfamiliar format. Moreover, as a high-profile event focused on failure, it was absolutely essential that the line-up of speakers be properly balanced in terms of gender, race/ethnicity, geography, and field of study, so that it would not be implied that scientific failure (or greatness!) was dominated by a particular group of people. In the end, the finalization of a line-up of six storytellers required approaching more than one hundred prestigious, selectively-targeted, scientists.

To prepare storytellers for the event, we provided guidelines adapted from The Moth (n.d.) for constructing a good story, with additional guidance on how to avoid some potentially dangerous pitfalls while telling personal stories in front of a professional audience (Box 1). We also provided the storytellers with examples of compelling storytelling from previous neuroscience events (The Moth Presents Wendy Suzuki, 2015) and met with each storyteller individually to discuss the outline for their stories.

Box 1. **Advice for Storytellers.** To prepare storytellers for the event, we provided guidelines adapted from The Moth (The Moth, n.d.) for constructing a good story, with additional guidance on how to avoid some potentially dangerous pitfalls while telling personal stories in front of a professional audience.

**What to do:**

 **Find your theme**

- Avoid linearity and long life-stories—focus on one central scene, one major event.

 **Have an arc—what are the stakes?**

- What did you stand to gain or lose? Why is what happened in the story important to you?
- Have a clear point of change in the story, end at a different point from where you started.

 **Have a great first line that sets up the stakes and grabs attention—start with the action!**

- Avoid starting by saying “this is a story about”, etc.
- No: “So I was thinking about climbing this mountain …”
- Yes: “The mountain loomed before me ….”

 **Show don't tell**

- Describe what happened as vividly as possible: What was the setting? (location, time?) Who was involved? How were things different at that point in your life than they are now?
- Avoid focusing on internal thoughts and explanations at the expense of action. Eloquent musings are great in writing but they won't feel like a story.

 **Steer clear of meandering endings**

- Have a strong clear final message or conclusion to your story.
- Your last line should be clear in your head before you start.

 **Keep it short and tight**

- Make critical choices—edit out redundant and unnecessary information.

 **Be yourself**

- Don't feel a need to act—part of the appeal of storytelling sessions is that they are genuine.
- Don't feel a need to be funny if you're not.

 **Know your story well enough so you can have fun!**

- Please know your story “by heart” but not by rote memorization. Notes, paper, or cheat sheets aren't allowed. Make an outline, memorize your bullet points, and play with the details.
- Enjoy yourself. Imagine you are at a dinner party, not a deposition.

**… and how to avoid potential pitfalls of storytelling within a professional setting:**

 **Your story about scientific failure should be your own.**

- Be careful how colleagues are mentioned—it may be worthwhile to anonymize cameos when possible or double-check that cameos are comfortable with their role/depiction in the story.

 **Some stones are best left unturned.**

- Be careful of stories that include legal culpability or violation of institutional ethics, especially in regard to animal or human subject research.

 **Please avoid fake accents**

- If your story doesn't work in your own voice, or that of your people of origin, please consider another story. Imitating accents from another culture or race rarely works and often offends.

 **Please practice civility and respect.**

- Avoid personal rants, as well as racism, homophobia, misogyny, or any form of hate speech.

### Impact

The storytelling proposal was accepted as a 50th anniversary event for SfN 2020. However, due to the pandemic, the event was rescheduled to 2021 and then, sadly, made remote. Despite this disruption, virtual attendance at the event numbered in the thousands and included a wide swath of the SfN population. There was a silver lining to the remote format—because the stories were pre-recorded, we were able to create a YouTube channel (Great Scientists, Great Failures, 2021b) and share the stories with a wider audience, receiving an additional ∼5,300 views and 250 subscribers. Once the channel was created, additional brave scientists were invited to record their stories, adding two more stories to the channel that have received >1,100 views.

### Overall assessment

This was unquestionably a high-effort, high-impact endeavor, requiring dedication on both the part of the organizers and the storytellers. As expected, we found that a high-profile storytelling session attracted a broad audience, providing a useful starting point for initiating conversations about failure. The stories within this particular event tended to focus on experiences with professional failure—failed collaborations, misguided mentorship, rejections, difficulties starting up a lab, and struggles with funding and self-worth. This emphasis meant that the stories were most useful for promoting resilience and mental health in the neuroscience community, and for serving as memorable professional training. Future storytelling events could focus on the science itself—failed replications, heavy criticisms, being scooped, experimental or theoretical mistakes—providing lessons in experimental troubleshooting, and encouraging the sort of scientific transparency necessary to improve research reproducibility and the revisions required for scientific progress.

We also found that the storytelling format was translatable to a remote setting, although video recordings lack the collective energy and immediate feedback received while performing in front of a live audience. This remote format allowed greater worldwide access, as well as potential use of the stories in classroom and training situations. That said, as an educational tool, these stories are powerful, but the audience's role is passive, especially within a remote format, and therefore we recommend providing space for facilitated post-event discussion.

## Interactive Game: “*Calculate Your F-Index*”

### Context

We developed the “*Calculate Your F-Index*” game as part of the interactive break-out session that we ran remotely over zoom immediately following the storytelling session at SfN 2021.This break-out session was intended to serve as a proxy for informal post-event conversation but ended up serving only a small number (<30) of participants. During the following 2 years (SfN 2022–2023), we ran the “*Calculate Your F-Index*” game in person as part of the SfN-sponsored social “*The Confound Hour: Let's Make Some Noise!*” (100–150 participants).

### Inspiration

We were inspired to create a game that reframed failures as accomplishments by the online purity tests popular in the late 1980s/1990s (“Wikipedia | Purity test,” 2025). Within these games, participants receive a point for everything that they have done wrong instead of what they have done right. We named the game “*Calculate Your F-Index*” as a satirical reference to the common H-Index metric used to evaluate research productivity.

### Vision

Although *F-Index* scores could be calculated independently by each individual, we designed the game to be run in a group setting, with each question presented on a slide, often with humorous visuals or cartoons. This upbeat, irreverent group context was generated to inspire public admission of fault—laughs, head nods, cheers—creating an atmosphere of solidarity that has some parallels with the bonding created by the common drinking game “*Never have I ever …*” (“Wikipedia | Never have I ever,” 2024).

### Logistics

Between 2021 and 2023, we generated two versions of the *F-Index* game: (1) The 2021 version was used in the remote break-out session for the “Oh Sh*t” storytelling session and focused on scientific failure, broadly speaking, with questions overviewing a variety of professional and experimental issues (Box 2). (2) The 2022–2023 version was used in person within the SfN-sponsored social “*The Confound Hour*” and had a narrower focus on experimental failure and scientific troubleshooting (Box 3).

Box 2. **“*Calculate Your F-Index*” (v. 2021).** This version of the *F-Index* game was used in the remote break-out session following the SfN 2021 “Oh Sh*t” storytelling session and focused on scientific failure, broadly speaking, with questions overviewing a variety of professional and experimental issues.

**Losing to Win: Calculate your F-Index *(1 point per yes)***

**Have you ever** **…**

1. Had a paper rejected?
2. … by more than 3 journals? (+1 point)
3. Failed a science class?
4. Overslept the morning of an important meeting or experiment?
5. Didn't realize that your camera or mic were on … at a rather critical moment?
6. Bombed a presentation?
7. … and it was your qualifying exam or dissertation defense? (+1 point)
8. Bombed your teaching evaluations?
9. … multiple semesters in a row? (+1 point)
10. Had more than 50,000 unread e-mails in your e-mail box?
11. Spilled a dangerous chemical or released a dangerous substance?
12. … and needed to use the safety shower or eyewash station? (+1 point)
13. … and had to evacuate or call Hazmat? (+1 point)
14. Had an animal escape and spent the day chasing it around the lab?
15. Missed a critical step in a protocol causing an expensive experiment to fail?
16. Accidentally destroyed a set of precious samples?
17. … and they weren't yours? (+1 point)
18. Labeled samples as “1–20” thinking you would *definitely remember* what they are…?
19. Invested in expensive equipment or supplies … and could never get it to work?
20. Lost data due to a hardware failure … and it wasn't backed up?
21. Hurt yourself in the lab?
22. … and had to go to the hospital? (+1 point)
23. Set fire to the lab?
24. Didn't tell anyone that it was you that ruined the thing?
25. Had a large experiment turn out to be confounded?
26. Had a manuscript that sat unfinished for more than 5 years?
27. … for more than 10 years? (+1 point)
28. Had a grant rejected?
29. … had 10 grants rejected? (+1 point)
30. … had 100 grants rejected? (+1 point)
31. … had to let students or staff go due to losing grant support? (+1 point).
32. Had a collaboration dissolve?
33. … and it was definitely your fault? (+1 point)
34. Been laid-off or fired?
35. … because a company that you started went under? (+1 point)
36. Had a journal tell you to have a native English speaker review your manuscript?
37. … and your native language is English? (+1 point)
38. Discovered errors in a paper *after* it was published?
39. … and had to submit an erratum? (+1 point)
40. … and had to retract the paper? (+1 point)
41. Had an irreproducible result?
42. … that you based your dissertation on? (+1 point)
43. … that you based your career on? (+1 point)
44. Cried in a lab or departmental office?
45. … and it was the office of the PI? (+1 point)
46. … and you are the PI? (+1 point)
47. Thought you made a mistake that was so big your career in science was over?
48. Thought about quitting science?
49. … daily? (+1 point)
50. …. But you're still here.

Box 3. **“*Calculate Your F-Index”* (v.2023).** This version of the *F-Index* game was used in person within the SfN-sponsored social “*The Confound Hour: Let's Make Some Noise!*” (2022 & 2023) and had a narrower focus on experimental failure and scientific troubleshooting.

**Losing to Win: Calculate Your F-Index**

**Cash in your troubleshooting woes for points!**

***(1 point per yes)***

**Have you ever** **…**

1. Spent more than a month trying to locate a source of electrical noise in a rig or recording set-up?
2. Had antibodies or probes label something other than the intended target?
3. Had small differences in a reagent that ended up making huge differences in the results?
4. Had an electrical or equipment failure mid-experiment and it changed the results in some important way?
5. Ran subjects on a task and then discovered that they were doing behaviors other than what the task was supposed to measure?
6. Had an experiment where your subjects simply refused to participate?
7.  … or maybe just died?
8. Discovered that your samples were contaminated or otherwise not what you expected them to be?
9. Discovered that something other than the target was altered by your genetic or drug manipulation?
10. … and just ran with it?
11. Had a result that turned out to actually be a bug in the code or computational error?
12. Gotten totally different results when using different normalization/analysis pipelines… and had no idea which version was correct?
13. Gotten completely different results when the experiment was run by different individuals or laboratories… and had no idea which version was correct?
  * +1 Extra point if it was actually yourself that failed to get the same result*
14. Inadvertently destroyed cells or tissue during the protocol that were supposed to remain healthy/intact?
15. Had something change in the surrounding environment that completely altered your subjects' behavior? (construction noise, pandemic!)
16. Had your experiment inadvertently change the exact phenomena that you were trying to study?
17. Discovered that a technical variable really, really mattered …

    … but that you didn't track it for most of your samples?

18. Had to change the protocol part way through the experiment because of funding issues?

**Lightning Round—Lab Accidents: 1 point for each!**

19.  Caused an explosion or set fire to the lab?

    … And had to call the Fire Department(+1 point)

20.  Flooded the lab?

    … And it affected more than one floor of the building? (+1 point)

21. Spilled a dangerous chemical or released a dangerous substance?

    … And had to use the eyewash station or safety shower? (+1 point)

    … And had to strip out of your clothes? (+1 point)

    … And had to evacuate and call Hazmat? (+1 point)

22. Hurt yourself in the lab and needed medical treatment?

   … And it happened in front of your PI? (+1 point)

  … And you're the PI. (+1 point)

We generated and vetted the questions included in the *F-index* games with input from social media, most extensively by vigorous discussion within the *Academic Research Moms* private Facebook group, and tailored the final list of questions to evenly encompass failures common across career stages and representing the many subfields in neuroscience (e.g., molecular, behavioral, neuroimaging, computational, electrophysiology). Importantly, the game was designed to reward points cumulatively for failures experienced over the course of a scientific career. This meant that the game was rigged—inevitably the top scorers would end up being the most senior (and therefore, probably the most successful) people in the room, simply because failure is a common and essential part of science, and senior scientists have had more time to fail repeatedly. This game mechanic was used to visually dissociate failure from youth and naiveté and ensure that the audience came away with the feeling that their own failures were not special, but both a common experience and essential for scientific success.

There were some key differences between running the game over zoom (<30 participants) and in person (100–150 participants per year). We found that keeping track of points was easier over zoom, where participants had easy access to some means of keeping notes. Within the in-person setting of an SfN-sponsored social, the game was dependent on access to a functional AV system and felt a little more chaotic, with some individuals reporting difficulty tracking scores or becoming confused after wandering into the social mid-game. We recommend that future facilitators invest in temporary access to anonymous smartphone interactive polling software for large groups, such as Poll Everywhere or Mentimeter.

At the end of the game, we provided a poll illustrating the distribution of points (over the years, we have only seen one 0!) and then asked the top scorers to reveal themselves. We have rewarded the winner in different ways—in person, this often took the form of drink tickets—accompanied by applause and cheers. We found that it was useful to further ensure that the winner was recognized for their insight and persistence by giving them with the opportunity to make a short “victory speech” offering words of wisdom for trainees *(“What advice would you give to younger scientists about overcoming failure? What helped you most?”*). In 2021, we have also used the *F-index* scores as a contributing criteria for winning the “*SfN “Stuck to It” Award*” and being coronated with a physical golden duct tape crown.

### Impact

In both remote and in-person contexts, the *F-index* game seemed to be an effective ice breaker, easing participants into admitting failure and quickly demonstrating that failure is common in science. In person, the game also created an upbeat atmosphere of solidarity and bonding.

### Overall assessment

The *F-index* game is relatively easy to run, in both remote and in-person contexts, with access to common tools (zoom, AV equipment, polling software). The questions that we used for a neuroscience audience (Boxes 2, 3) could be easily adapted to other scientific fields. Most notably, the game scales effectively—it is as easy to run the game in a small group as in a very large group. That said, the game lacks the depth of empathy generated by detailed storytelling or confessionals and may be best paired with facilitated discussion.

## Interactive Game: Science Failure *BINGO*

### Context

We developed Science Failure *BINGO* as part of the in-person SfN-sponsored social “*The Confound Hour: Let's Make Some Noise!*” at the 2024 annual SfN conference (∼50 participants).

### Inspiration

We found that the *F-index* game was good at getting participants warmed up and excited to share stories about scientific failure, but it had the downside of being mostly noninteractive. To better encourage interaction, in 2024 we adapted a common icebreaker game Human *BINGO* (wikiHow, n.d.) to include a wide assortment of scientific woes (e.g., confounds, artifacts, lab accidents, disastrous mistakes, scientific fiascos, etc.).

### Vision

The goal was to encourage participants to mingle and find people who had experienced the assorted scientific woes listed on their *BINGO* cards. Participants were allowed to only mark off one box per person, forcing them to meet and talk with each other. The first participant to mark five boxes in a row (horizontal, vertical, or diagonal) would then shout “BINGO!” and win a drink ticket. Our hope was that the game would turn the norms of conference networking solidly on their head, as the game quickly makes the individuals in the room who are the most open about their failures the most socially valuable. Following the *BINGO* win, we also allowed participants to cash in on their own woes by giving themselves 1 point per “Yes” for all of the struggles that they had personally experienced on their own cards. The participant with the highest score was then declared a winner and given a drink ticket, allowing participants to celebrate their own failures as well as those of their peers.

### Logistics

**Figure 1.**
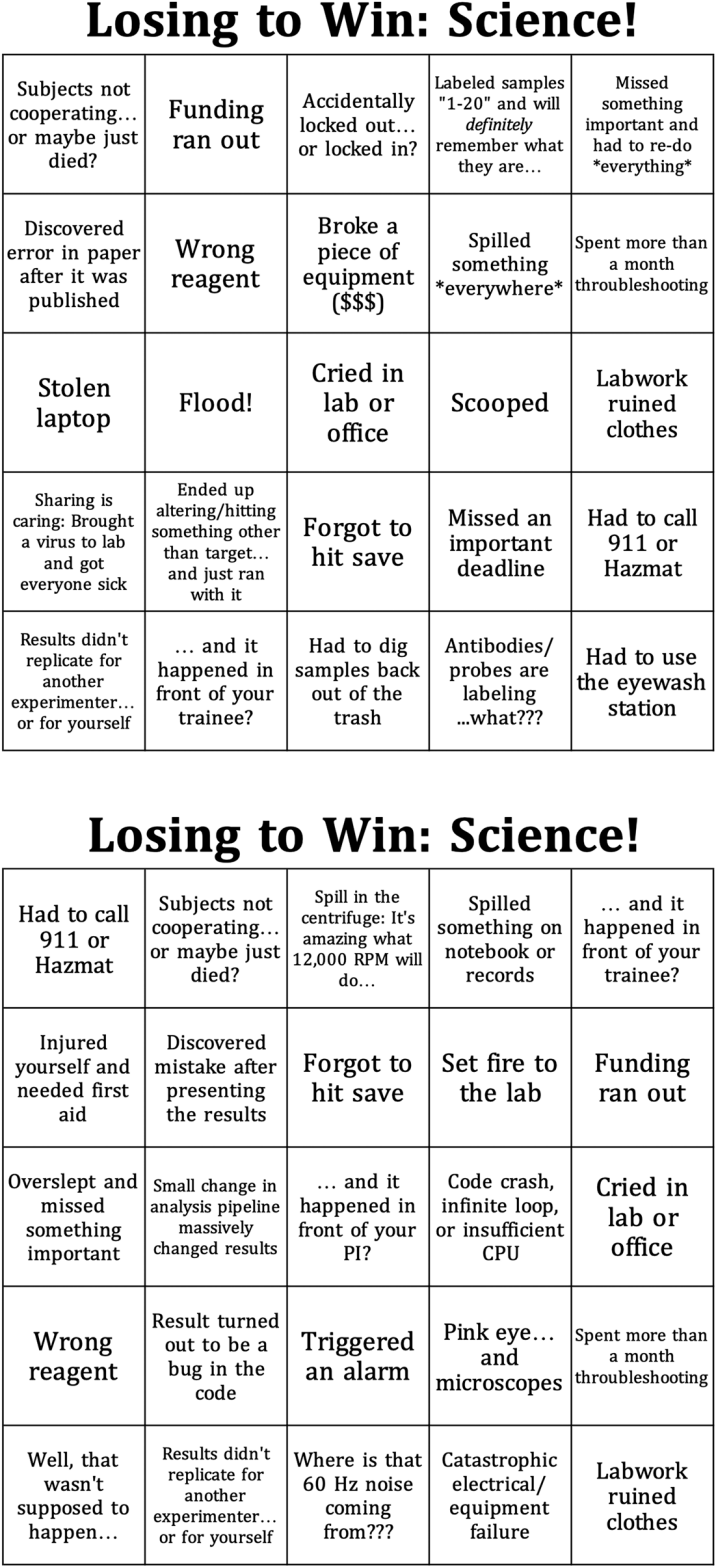
Failure *BINGO*. Example *BINGO* cards resembling those used in the in-person SfN-sponsored social “*The Confound Hour: Let's Make Some Noise!”* (2024). A full set of *BINGO* cards can be generated and downloaded for free online (“myfreebingocards.com,” n.d.)

To create the *BINGO* cards, we adapted the content of the *F-index* game to an abbreviated format, making sure that there was a decent mixture of low-hanging fruit (“*Cried in lab or office”*), common mistakes *(“Result turned out to be a bug in the code”, “Spill in the centrifuge: It's amazing what 12,000 RPM will do …”*), and rarer catastrophes *(“Set fire to the lab”, “Stolen laptop*”), using humor when possible to maintain an upbeat atmosphere *(“Spilled radioactivity, no superpowers yet”, “Labeled samples 1–20 and will definitely remember what they are”*). We then used an online *BINGO* card generator to generate a set of 30 unique cards from our list of scientific woes (Fig. 1, a full set of the scientific failure *BINGO* cards can be generated and downloaded for free; myfreebingocards.com, n.d.) and printed multiple sets of the 30 cards with the assumption that the randomness of mingling would ensure that the experience of each participant filling out a card was unique. We also brought extra pens to the event but found that most participants had their own writing utensils.

### Impact

We distributed the *BINGO* cards when participants arrived at the door for the social and found that even before the game began, the cards were inspiring conversation. Once the game officially began, the room erupted into busy enthusiastic mingling. The game was actually so successful at encouraging interaction that the room was too loud to hear folks shout *BINGO*—we ended up having six people win *BINGO* before we were able to gain the attention of the full group to switch to the next activity!

### Overall assessment

The game was easy to run, did not require AV equipment, and was effective at building rapport between participants and creating an upbeat atmosphere. The prompts that we used for a neuroscience audience could be easily adapted to other scientific fields. We only ran the game with approximately 50 participants, but the game would likely scale effectively to a larger crowd. The only downside was the duration—altogether, the game only lasted 10–15 min. The game could potentially be expanded by using *BINGO* cards larger than a 5 × 5 grid, replacing some of the low-hanging fruit among the answers *(“Thought about quitting science”)* with less common struggles, or by distributing a second set of *BINGO* cards.

## Confessionals: Individual and Team Based

### Context

We featured confessionals as a core part of each of our four interactive events hosted within the 2021–2024 annual SfN conferences, including the remote “Oh Sh*t” *Break-out Session* during SfN 2021 and the in-person social “*The Confound Hour: Let's Make Some Noise!*” at SfN 2022–2024.

### Inspiration

Following the complicated logistics of organizing the formal storytelling session, we were impressed by how freely participants in the post-event break-out session shared stories about their own experiences with failure. Of course, these spontaneous confessionals occurred after famous scientists had just modeled sharing vulnerability in a professional setting and also followed participation in the *F-index* game, but they implied that open discussion of failure might be easily promoted within a spontaneous, low-stakes, supportive environment. We tested out this hypothesis during the next 3 years, within the format of an in-person SfN-sponsored social (“*The Confound Hour*”).

### Vision

We billed the social as an entertaining opportunity for scientists to discuss work that didn't make it to the main floor of SfN (*“Did you have an experiment that tanked this year? Now is your time to shine!”*). At the event, the audience was encouraged to cheer loudly for each confessional, in empathy and solidarity, celebrating the storyteller's insight and persistence.

### Logistics

We tested out multiple formats for spontaneous confessionals. Within the remote break-out session following the storytelling session (SfN 2021), we had participants meet in small groups (zoom break-out rooms) to brainstorm ideas (*“You deserved more points: What failures should have been part of the F-Index but weren't?”, “Would you be willing to be a storyteller? If so, what story would you tell?”*), and then rejoin the group to share their stories and ideas either verbally or in the chat.

At the in-person social at SfN 2022–2024 (*“The Confound Hour”*), we started with a game (*F-index* or *BINGO*) to create a rowdy, upbeat atmosphere. In 2022, we then had participants volunteer to come up on stage and share their stories, with the first 10 volunteers receiving drink tickets. For each confessional, we encouraged the audience to cheer loudly. To avoid potential legal or professional pitfalls in a spontaneous live event, we provided a set of rules for participants (Box 4). We recommend that future facilitators leave these rules posted during the confessionals and occasionally provide reminders, as it was awkward to navigate providing a supportive (rowdy!) environment for confessionals while responding to stories about mistakes with potential legal/ethical ramifications. For example, although it is important for trainees to have opportunities to discuss failures in animal research to prevent reoccurrence, publicly discussing any mistakes that involve real distress to the animals is risky in our current polarized climate. Likewise, it is absolutely essential that confessionals remain personal, with any cameos from colleagues anonymized to avoid professional backlash.

Box 4. **Rules of Conduct for Confessionals.** To avoid potential legal or professional pitfalls in a spontaneous live event, we provided a set of rules for participants in “*The Confound Hour*.” We recommend that future facilitators leave these rules posted during the confessionals and occasionally provide reminders, as it was awkward to navigate providing a supportive (rowdy!) environment for confessionals while responding to mistakes with potential legal/ethical ramifications.

**Rules of Conduct:**

- **This is a supportive environment: We're here to build each other up.** If you want to tear people down, go hang out on Twitter instead.
- **If you hear a good story here, think twice before passing it on.** Get permission or anonymize.
- **Your stories should be your own.** Be careful how colleagues are mentioned—it may be worthwhile to anonymize cameos.
- **Some stones are best left unturned.** Be careful of stories that include legal culpability or violation of institutional ethics, especially in regard to animal or human subject research.
- **Please practice civility and respect.** Avoid personal rants, as well as racism, homophobia, misogyny, ethnic slurs, or any form of hate speech.
- **Harassment & General Assholery:** If you need help, let the host know..

In 2023–2024, we had participants break up into teams (6–10 individuals) and then structured the confessionals as a team-based competition following the format of the game *Two Truths and a Lie* (wikiHow, n.d.). Each team was given time (20–25 min) to come up with their best stories of experimental failure, as well as one fake story. We then went around the room: in each round, a representative from one team presented their three stories, and the remaining teams voted on which story was the lie. The lie was revealed, and the voting teams received points if they were correct, whereas the presenting team received points for each incorrect answer. After the reveal, audience members could demand further details about the true stories. In the future, we recommend that facilitators bring some method for publicly tracking team points (e.g., white board or large pad, projected excel sheet) and a portable microphone. The game concluded with an announcement of the winning team, as well as audience nomination and vote for the best stories. In 2022–2024, the best storyteller was coronated with a physical golden duct tape crown and awarded the “*SfN “Stuck to It” Award*” in celebration of their persistence in the face of adversity.

### Impact

Over the past 4 years, our interactive events, which included both the *F-index* and *BINGO* games and spontaneous confessionals, have been attended by several hundred participants. In comparison to the formal storytelling session, these events have typically attracted a younger crowd—mostly students and post-docs—representing a diverse variety of backgrounds (genders, race/ethnicity, institutions, countries of origin).

Although the long-term effects of the events on scientific practice are unknown, at each event there was an immediate, visible impact on transparency, appreciation of failure, and mental health. Within the sessions, there is a palpable feeling of collective catharsis and visible bonding, with newly formed groups of participants leaving the sessions together. Afterward, participants told us that they felt less alone, more confident, more connected, or even “healed.” As one participant recently wrote “I initially felt a bit out of place at the conference, but your social hour provided an excellent platform for meaningful conversations among scientists. It was truly enjoyable and sparked insightful discussions that I carried back to my lab.” In a post-session survey, participants unanimously reported that our event made them feel like failure is a regular part of successful science, and they, in turn, viewed their own failures in a more positive light.

### Overall assessment

Logistically, interactive events encouraging spontaneous confessionals seem to provide the greatest impact in proportion to the effort spent organizing them. These events worked well over a remote platform (zoom) and in person and were even feasible in a crowd of >100 with broken AV equipment. The downside is a restricted ability to scale the events—the larger the group, the harder it is to create the sort of “low stakes” atmosphere that encourages sharing stories about failure. We found that team-based activities helped encourage more active participation, allowing anxious or introverted individuals to contribute to smaller scale discussions, while extroverted individuals represented the team's stories to the larger audience. That said, even within the less structured “take the stage” format of 2022, we still had more than 25 people spontaneously share confessionals to an audience of over 100. Thematically, the use of the format of Two Truths and a Lie appears to fit well with the theme of confessions—in order to make a true story appear as a lie it should be somewhat outrageous or have some dramatic aspects—this motivates participants to share more of their “bigger” stories.

To scale up further, it might be possible to have individuals or teams share their best stories over an interactive online platform and then have voting take place online. For example, in parallel at SfN 2022, *The Community for Rigor*, an NIH-funded educational initiative, collected confessionals from attendees at their booth and via an online submission portal, focusing specifically on the ways that attendees had grown in terms of scientific rigor, including challenges related to *p*-hacking, experiment preregistration, and lab workplace dynamics. These submissions were then shared anonymously online for the next 6 months on the organization's Twitter account (@comm4rigor) following screening for inappropriate or injurious content. Similar to our experiences within “*The Confound Hour”*, *Community for Rigor* found that both the in-person interviews at their booth and online confessionals served as a rich, generative means for understanding the shape and scale of the problems that arise when science only talks about success.

## Moving Forward

There has been strong support for creating space within the annual SfN meeting for open conversations about scientific failure. Behind the scenes, more than 150 scientists contributed to the creation of the storytelling session, virtual break-out session, YouTube Channel, and socials, providing suggestions, networking, encouragement, and feedback. Within the events, more than 60 scientists were willing to stand in front of a crowd of their peers and break through a wall of stigma with their openness and honesty, sometimes with great trepidation. We have received enthusiastic feedback from trainees, as well as patronage from senior members within the SfN organization.

On a structural level, SfN enabled the creation of these events by making formal storytelling sessions a featured event at the annual SfN conference. This emphasis on humanizing science was essential for paving the way for both our failure-focused storytelling event and failure-focused SfN-sponsored socials. For other organizations that might wish to follow suit, the introduction of storytelling sessions into the annual conference was aided by collaborations with StoryCollider (“The Story Collider,” 2025), which is a non-profit organization dedicated to sharing stories from STEM professionals in live shows and podcasts. StoryCollider provides both workshops and direct consulting to facilitate the creation of storytelling events on campuses, workplaces, and conferences.

Even with this exceptional support, we found that organizing failure-focused events presented an unusual set of challenges. The recruitment of formal speakers is particularly tricky, as discussions of scientific failure are typically conducted outside of the public spotlight. A prospective failure-focused session organizer cannot easily scan faculty bios for evidence of struggle, blunder, and rejection or skim recent abstracts for descriptions of confounds, failed experiments, and lab accidents. This can limit potential speakers to scientists within the immediate network of the prospective organizer, or to individuals who happen to respond to e-mail solicitations or blind queries posted on social media. At best, this produces proposals lacking in desired diversity; at worst it produces a complete dead end. For example, “*The Confound Hour*” was originally conceived to be a data blitz for confounds, artifacts, and failed experiments, but recruitment via networking and social media proved to be too difficult. In this respect, formal storytelling events may be easier to organize within smaller, tight-knit scientific communities. Within larger scientific organizations, these events could be greatly facilitated by central support to broadly invite participation.

We have emphasized storytelling and society-sponsored socials as fruitful media for encouraging public discourse about scientific failure, but there are many other ways that scientific organizations could recognize the everyday, essential presence of failure woven into our scientific pursuits. For example, we present scientific awards for groundbreaking scientific accomplishments, teaching, mentorship, and outreach—what if we committed to featuring stories of professional struggle and not just showcasing success? What if we invited scientists to provide high-profile lectures trumpeting their mistakes and revisions as much as their accuracies—to openly discuss the downfall of their favorite theory, the biases and fallacies that led them astray? What if we provided awards for transparency and open science and not just achievement? At conferences, how much could we accelerate the pace of science if we invited members to submit abstracts for poster sessions dedicated to experimental troubleshooting as well as results—challenging artifacts, confounds, logistical fiascos, conflicting results, issues with reproducibility and replication?

## Conclusion

Over the years, our efforts have received legitimate criticism for our use of the term “failure.” Is it a failure to struggle, to balk in the face of extraordinary setbacks, to have ideas vetted and rejected, to lose repeatedly in order to learn? Should we describe the mistakes and troubleshooting essential to scientific discovery as failure? The unearthing and revision of faulty ideas? Isn't this just science? Yes it is. Together, let us decrease the stigma surrounding “failure” and commit to legitimizing these experiences by bringing them out of the shadows and into the main program of our scientific conferences.

## Synthesis

Reviewing Editor: Nathalie Ginovart, University of Geneva Decisions are customarily a result of the Reviewing Editor and the peer reviewers coming together and discussing their recommendations until a consensus is reached. When revisions are invited, a fact-based synthesis statement explaining their decision and outlining what is needed to prepare a revision will be listed below. The following reviewer(s) agreed to reveal their identity: Nicola Simcock.

Both reviewers acknowledged the article's value in promoting a more open discussion about failure in scientific research. They support the idea that failure is an essential part of the learning process and appreciate the broad approach taken by the authors, suggesting that the proposed activities could foster better engagement and enhance transparency in neuroscience.

Please find the specific comments raised by the reviewers below:

Reviewer 1:

The objective of the article is not to advance the field but rather propose a forum in the SfN meeting that could be applied to over venues.

This is a well-articulated article, and I commend the authors for their good intentions. That said, the narrative suffers from a basic lack of definition of what a "failure" is. While successes often remain, failures come and go. Some failures are for good reasons and one can build upon, whereas others are simply not that insightful. As the authors will acknowledge, most often a string of failures is followed by successes, as successes help us define what a failure is. The authors aim to engage scientists to talk about their failures, but the implicit expectation is that participating scientists should have been successful at some point so that their stories of failure would be encouraging and illuminating. Taken together, I find the overall argument somewhat flawed. My specific comments are below:

1. Page 4: 'But doing so also encourages the imperfection and complexity inherent to science to be buried within carefully worded results and supplemental material, inaccurately representing the scientific process and precluding self-correction. If our field is to move beyond the reproducibility crisis, we need greater transparency, and with that, we need to normalize the admission of failure'. How do carefully written results misrepresent the scientific process and contribute to reproducibility crisis? Often there are many layers of self-correction that go into a manuscript as a paper is being put together. In addition, in well-organized papers negative results go hand in hand with positive results, as they increase our confidence of the veracity of positive results. A "failure" today, may simply be an excellent negative control of tomorrow.

Page 4: is there data to support public discussions of scientific failure promote mental health? Later in this paragraph 'failure...causing isolation and depression'. Again, is there data that scientific failure causes depression, a serious mental disorder?

Page 20: The authors suggest providing awards for transparency and open science and not just achievement. How would one go about selecting a 'winner'? A common complaint is that the same people keep winning awards regardless of the metrics, which itself can be demoralizing. Social media also has many caveats as some of the most vocal voices may not necessarily be the best representatives of a topic or a field.

Page 20: How do the authors envision their suggestion of submitting abstracts for poster session dedicated to experimental troubleshooting? Poster abstracts are often quite preliminary with data continuing to be collected with results being further defined.

Overall, SfN can definitely incorporate more public interviews and discussions with senior scientists - who have been successful in something at some point - to open up regarding their career trajectories as well as "failures" but events solely focusing on failures may simply be hard to quality control and may fail to generate enthusiasm.

Reviewer 2:

This paper explores the topic of failure in relation to Neuroscience (specifically the annual Society for Neuroscience conference) and the importance of talking more openly about this subject. As the author(s) highlight, failure is an under-discussed topic and one that can have a huge impact on all members of the research community, from improved scientific rigour to enhanced mental wellbeing among others. Sharing stories of failure in research is rare, and promoting innovative ways to do this for a diverse audience will be of great value to the field and beyond. The impacts of sharing these ideas are wide ranging and the author(s) detail a useful example in the introduction about how the 'infallibility of science' can cause science communication issues in the public domain, which reinforces the need to 'get this right' within field, to allow broader improvements.

The paper provides an excellent set of resources that are useful to neuroscientists and many others. The activities outlined, and learnings from previously held sessions, offer usable templates for readers to employ in their own areas, both online and in-person allowing adaptability and promoting accessibility. The paper makes a great comparison of how the activities are relevant to different cohorts and considers the inputs and outcomes for senior academics as well as junior colleagues. These innovative approaches will help to increase and maintain diversity and longevity in the field.

This paper is a wonderful presentation of innovative practices to promote the often-overlooked topic of failure. Thank you for trialling these activities and sharing them openly for others to benefit from. It would have been good to include more feedback from conference participants, but appreciate that you may not have this data.

## References

[B3] Great Scientists, Great Failures: Dr. Stuart Firestein (2021a) Available at: https://youtu.be/9r3s-54pxPg?si=4k1wtOASR6WSbyU-.

[B4] Great Scientists, Great Failures: Playlist (2021b) Available at: https://youtube.com/playlist?list=PLejoG7h0KXWJhvDRKoc0euANaYv6e3GuX&si=e6VoYjKvAsbMIKIX.

[B7] myfreebingocards.com - free custom bingo card generator [WWW Document] (n.d.) myfreebingocards.com. Available at: https://myfreebingocards.com/bingo-card-generator (Accessed 2.12.25).

[B11] The Moth (n.d.) Storytelling Tips & Tricks [WWW Document]. The Moth (en-US). Available at: https://themoth.org/share-your-story/storytelling-tips-tricks (Accessed 2.12.25).

[B1] The Moth Presents Wendy Suzuki: Saying “I Love You” (2015). The Moth. Available at: https://youtu.be/GMCNOGCgTfg?si=1BITDyKaUSYOz67h (Accessed 2.12.25).

[B2] The Moth (n.d.) The Art and Craft of Storytelling [WWW Document]. The Moth (en-US). Available at: https://themoth.org/ (Accessed 2.12.25).

[B10] The Story Collider [WWW Document] (2025) The Story Collider. Available at: https://www.storycollider.org (Accessed 2.12.25).

[B5] wikiHow (n.d.) How to Play Human Bingo [WWW Document]. wikiHow. Available at: https://www.wikihow.com/Play-Human-Bingo (Accessed 2.12.25).

[B6] wikiHow (n.d.) How to Play Two Truths, One Lie [WWW Document]. wikiHow. Available at: https://www.wikihow.com/Play-Two-Truths,-One-Lie (Accessed 2.12.25).

[B8] Wikipedia (2024) Never have I ever. Wikipedia.

[B9] Wikipedia (2025) Purity test. Wikipedia.

